# Efficacy and safety of rilonacept for recurrent pericarditis: results from a phase II clinical trial

**DOI:** 10.1136/heartjnl-2020-317928

**Published:** 2020-11-23

**Authors:** Allan L Klein, David Lin, Paul C Cremer, Saifullah Nasir, Sushil Allen Luis, Antonio Abbate, Andrew Ertel, Martin LeWinter, Anna Beutler, Fang Fang, John F Paolini

**Affiliations:** 1 Center for the Diagnosis and Treatment of Pericardial Diseases, Section of Cardiovascular Imaging, Department of Cardiovascular Medicine, Heart, Vascular, and Thoracic Institute, Cleveland Clinic, Cleveland, Ohio, USA; 2 The Minneapolis HeartInstitute, Abbott Northwestern Hospital, Minneapolis, Minnesota, USA; 3 Stat! Cardiology, Chicago, Illinois, USA; 4 Division of Cardiovascular Ultrasound, Department of Cardiovascular Medicine, Rochester, Minnesota, USA; 5 Pauley Heart Center, Virginia Commonwealth University, Richmond, Virginia, USA; 6 Heart and Vascular Institute, Medstar Washington Hospital Center, Washington, DC, USA; 7 Cardiology Unit, University of Vermont Medical Center, Burlington, Vermont, USA; 8 Kiniksa Pharmaceuticals Ltd, Hamilton, Bermuda; 9 Kiniksa Pharmaceuticals Corp, Lexington, Massachusetts, USA

**Keywords:** pericardial disease, inflammatory markers, pericarditis

## Abstract

**Objective:**

Recurrent pericarditis (RP) incurs significant morbidity. Rilonacept inhibits both interleukin-1 alpha (IL-1α) and IL-1β; these cytokines are thought to play a major role in RP. This phase II study evaluated rilonacept efficacy and safety in RP.

**Methods:**

This multicentre, open-label study enrolled adult patients with idiopathic or postpericardiotomy RP, symptomatic (≥2 pericarditis recurrences) or corticosteroid (CS) dependent (≥2 recurrences prior).

Patients received rilonacept 320 mg SC load/160 mg SC weekly maintenance in a 6-week base treatment period (TP) followed by an optional 18-week on-treatment extension period (EP) (option to wean background therapy).

**Results:**

Outcomes: pericarditis pain (numeric rating scale (NRS)) and inflammation (C reactive protein (CRP)) for symptomatic patients; disease activity after CS taper for CS-dependent patients. Secondary outcomes: health-related quality of life (HRQOL), pericarditis manifestations and additional medications. 25 unique patients enrolled, while 23 completed the EP (seven colchicine failures and five CS failures). In symptomatic patients, NRS and CRP decreased; response was observed after first rilonacept dose. NRS decreased from 4.5 at baseline to 0.7, and CRP decreased from 4.62 mg/dL at baseline to 0.38 mg/dL at end of TP. Median time to CRP normalisation: 9 days. Pericarditis manifestations resolved. 13 patients on CS at baseline completed the EP; 11 (84.6%) discontinued CS, and 2 tapered; CRP and NRS remained low without recurrence. Mean HRQOL scores improved in symptomatic patients. One serious adverse event (SAE) resulted in discontinuation of rilonacept.

**Conclusions:**

Rilonacept led to rapid and sustained improvement in pain, inflammation (CRP and pericarditis manifestations) and HRQOL. CSs were successfully tapered or discontinued; safety was consistent with known rilonacept safety profile.

**Trial registration number:**

NCT03980522.

## Introduction

Recurrent pericarditis (RP) is associated with debilitating chest pain, physical limitations, decreased quality of life and emergency department visits and hospitalisations.^1^

It frequently occurs following a first episode of acute pericarditis, with reappearance of pericarditis signs and symptoms after a symptom-free period of at least 4–6 weeks^2^ and affects 15%–30% of patients.^1^ The chance of future recurrences increases with each additional recurrence,^3^ and among patients with two or more recurrences, the probability of further recurrence is 20%–40%.^4^

The mainstay of current treatments includes non-steroidal anti-inflammatory drugs (NSAIDs), colchicine and corticosteroids (CS). For colchicine-resistant and CS-dependent RP, other immunomodulatory treatments or surgical pericardiectomy are considered, though with limited data.

The broad immunosuppression and side effects of CS call for more targeted therapies in RP. While the mechanisms underlying RP are not fully elucidated, an autoinflammatory response characterised by inappropriate activation of the innate immune system, in particular the interleukin (IL) 1 family of cytokines, has been implicated. IL-1 is the primary proinflammatory cytokine responsible for autoinflammatory disorders, including RP,^5^ and therefore, blocking IL-1 activity was hypothesised to provide therapeutic benefit in RP. Anakinra, a recombinant IL-1 receptor antagonist, was previously evaluated in an investigator-initiated placebo-controlled study in a small number of CS-dependent colchicine-resistant patients with idiopathic RP, many of whom continued therapy with colchicine during the study, as well as in a registry in a ‘real world’ population.^6 7^

Rilonacept, approved in the USA for the treatment of cryopyrin-associated periodic syndromes (CAPS)^8^ (Arcalyst; Regeneron, Tarrytown, New York, USA), inhibits IL-1 by binding to IL-1α and IL-1β.^8 9^ Given the autoinflammatory pathobiology of RP and the mechanism of action of rilonacept with its dual IL-1 blockade, we hypothesised that rilonacept would be an effective novel therapy in RP and conducted a pilot study in a broader population of RP patients to assess resolution of pericarditis symptoms, improvement in objective measures of disease, feasibility of weaning CS in CS-dependent patients and safety.

## Methods

### Data sharing statement

The individual anonymized data supporting the analyses contained in the manuscript will be made available upon reasonable written request from researchers whose proposed use of the data for a specific purpose has been approved.

### Patient and public involvement

Patients or the public were not involved in the design, or conduct, or reporting, or dissemination plans of our research.

### Study design

**Figure 1:**
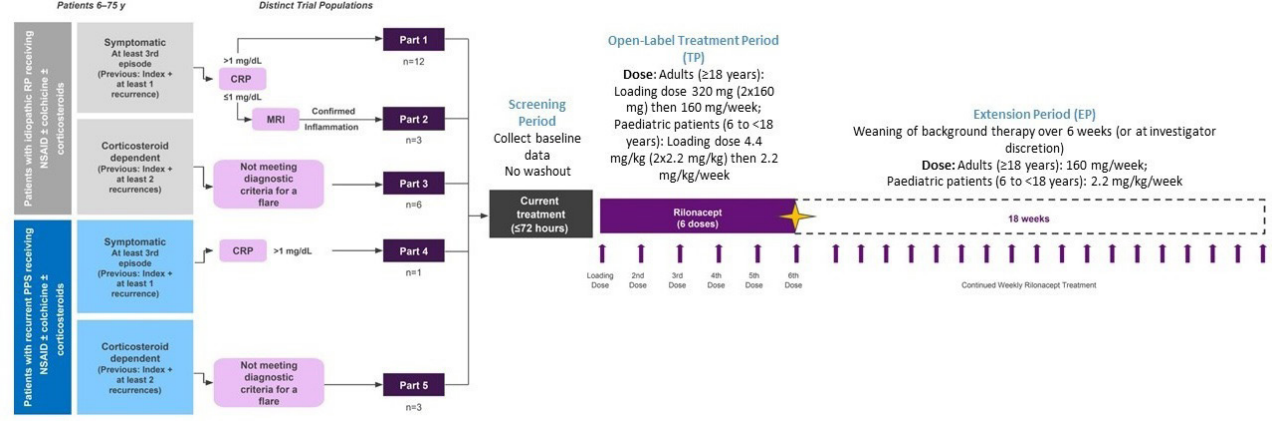
Study design.

This open-label, single-active-arm, five-part, phase II study evaluated the efficacy and safety of rilonacept in patients with RP, explored clinical and biochemical endpoints and collected interpatient and intrapatient variability data (figure 1). The study was conducted between January 2018 and May 2019 at nine sites in the USA.

### Patient populations and study parts

Eligible patients were adults (18–75 years) or children (≥6–<18 years) with RP due to idiopathic or postpericardiotomy aetiology, presenting with at least a second recurrence of pericarditis or, in the absence of an active recurrence, at least two recurrences previously and CS dependence at time of enrolment. Thus, two specific patient populations were included: those with a symptomatic episode and signs of inflammation (active recurrence) and those without active recurrence but with CS-dependent disease. Specific inclusion criteria by study part were as follows (figure 1): patients with active disease had to present during a symptomatic RP episode, having experienced a first episode of pericarditis and at least one recurrent episode before the enrolment-qualifying event, either of idiopathic (parts 1 and 2) or postpericardiotomy syndrome (PPS) aetiology (part 4). Parts 1 and 4 patients had to have a C reactive protein (CRP) value at screening >1 mg/dL, while part 2 patients had CRP ≤1 mg/dL attributed to concomitant medications (eg, CS) and evidence of pericardial inflammation assessed by delayed pericardial hyperenhancement on cardiac MRI. CS-dependent patients (parts 3 (idiopathic) and 5 (PPS)) had to present with CS-dependent disease, with a first episode of acute pericarditis followed by at least two recurrent episodes and without active pericarditis at time of screening. CS dependency was based on investigator judgement and defined as anticipated return of signs and symptoms of pericarditis based on previous attempts at CS tapering. For all study parts, episodes of pericarditis were defined using the 2015 European Society of Cardiology (ESC) Guidelines for the Diagnosis and Management of Pericardial Diseases as a framework.^2^

At study entry, patients in all parts were allowed concomitant NSAIDs, and/or colchicine, and/or CS (in any combination) if the dosages had been stable for ≥7 days; patients in parts 3 and 5 were required to be taking CS at enrolment. All patients gave written informed consent.

### Treatment and procedures

In the base Treatment Period (TP) eligible adults received a loading dose of 320 mg rilonacept (KPL-914), administered via subcutaneous (SC) injection on day 0, followed by 160 mg SC weekly for 5 additional doses; dose and administration schedules were consistent with the Food and Drug Administration (FDA)-approved schedules for rilonacept in CAPS.^8^ Conventional concomitant pericarditis medications (eg, NSAIDs, colchicine and/or CS) were maintained at prestudy dose levels, not to be changed unless medically indicated throughout the 6-week base TP. In the optional 18-week treatment extension period (EP), during which rilonacept weekly injections continued, investigators were given the option to wean their patients from concomitant medications as follows: for NSAIDs and colchicine to taper within 15 days of EP entry, and for CS to taper by 5 mg prednisone or equivalent each week in adults so as to withdraw within 6 weeks of EP entry.

### Efficacy assessments

For active pericarditis patients (parts 1, 2 and 4), the primary efficacy endpoints were patient-reported pericarditis pain using an 11-point pain numeric rating scale (NRS), validated across multiple conditions with acute and chronic pain^10–12^ and CRP at baseline and on-treatment. For CS-dependent patients (parts 3 and 5), the primary efficacy endpoint was disease activity after tapering CS. Across all parts, secondary endpoints were improvement in pericarditis manifestations other than pain and CRP, change in patients’ health-related quality of life (HRQOL) using the validated Patient-Reported Outcomes Measurement Information System (PROMIS) questionnaire (V.1.2-Global Health) to assess overall physical and mental well-being,^13^ use of concomitant CS and changes in the use of other concomitant medications for pericarditis.

Detailed descriptions of assessment of cardiac MRI, pharmacokinetics and antidrug antibody (ADA) are provided in online supplemental methods.

10.1136/heartjnl-2020-317928.supp1 Supplementary data

### Statistical analysis

Because of the small sample size and study design, no inferential statistical analyses or hierarchical testing were planned. For continuous variables (eg, change from baseline), summary statistics were calculated as means (SD) and medians (ranges). For categorical variables, summary statistics were calculated for each part. All analyses are based on observed data.

Values are presented as means (SD), and/or medians (range) if only medians were available or if medians were different from means.

Select analyses were performed for pooled groups of parts 1, 2 and 4 (parts enrolling subjects during symptomatic episode) and parts 3 and 5 (parts enrolling subjects without active episode but CS dependent) for greater statistical precision in determining therapeutic response or safety.

All analyses were performed without imputation for missing data and were conducted using SAS V.9.4.

In order to evaluate the effect of rilonacept treatment on pericarditis recurrence, data collected included the number of pericarditis episodes at enrolment (index, prior recurrences and current episode) and the number of episodes during the study (recurrences during base TP and EP combined). The annualised incidence of pericarditis episodes was assessed before and after treatment with rilonacept. Annualised incidence prior to the study was calculated by dividing the number of pericarditis episodes at enrolment (including index, recurrences and qualifying episode) by disease duration in years. Annualised incidence of pericarditis episodes during the study was calculated by dividing the number of recurrences during the study by study duration in years.

### Safety assessments

Treatment-emergent adverse events (TEAEs; defined as Adverse events (AEs) reported after the first study drug administration) were recorded by investigators, including level of severity (mild, moderate or severe) and relationship to study drug (not related, unlikely related, possibly related or related). Serious TEAEs were defined as events that were life threatening or resulted in death, or required hospital admission or prolonged hospitalisation, or resulted in persistent or significant disability/incapacity, or important medical events that jeopardised patients or required an intervention to prevent a serious outcome. Safety clinical laboratory testing included local haematology, chemistry, urinalysis and central laboratory lipid panel. Physical examinations included vital signs, weight and height.

## Results

### Patient disposition and demographics

Among 49 patients screened, 25 unique adult patients were enrolled and received rilonacept (online supplemental figure 1). No paediatric patients were enrolled. One patient participated in the study twice: this patient successfully completed the study but, approximately 11 weeks after rilonacept discontinuation, developed a pericarditis recurrence (pain NRS 7/10; CRP 23.1 mg/dL) and cardiac tamponade requiring pericardiocentesis. The patient was re-enrolled in the study as the 26th patient while study enrolment was still open and subsequently had resolution of pericarditis and an uneventful clinical course. Assessments from this patient’s first participation only are included in the analysis. All 25 patients were analysed for the primary efficacy endpoints and for safety. Of 23 patients who entered the EP, 23 (100%) completed it; one CS-dependent PPS patient (part 3) completed the base TP but declined to continue into the EP, and one symptomatic idiopathic RP patient (part 1) experienced a serious AE and discontinued the study drug after visit 4 in the base TP (discussed further).

Patient demographics were generally similar across study parts. Among patients entering the base TP, mean age was 42.8 years (range 26–62 years), 60% of patients were female and the majority of patients were white (table 1). Mean baseline NRS pain scores ranged from 4.0 to 4.7 for symptomatic patients (parts 1, 2 and 4), and mean baseline NRS pain scores were 1.2 and 2.0 for CS-dependent patients (parts 3 and 5, respectively). At study entry, the majority of patients (~80%) were taking two or more medications for their pericarditis. The mean (median; range) number of pre-enrolment pericarditis recurrences was 2.6 (2; 1–8), and the annualised incidence of pericarditis episodes (including index, recurrences, and qualifying episodes, if applicable) prior to study entry was 3.9 (2.5; 0.5–15).

**Table 1:** Baseline demographic and clinical characteristics

Disease status: CRP requirement (mg/dL): n:	Idiopathic			PPS		Total
	Active* >1 12	Active† ≤1 3	CS-dep‡ N/A 6	Active§ >1 1	CS-dep¶ N/A 3	All*†‡§¶ N/A 25
Mean (median, range, SD) age, years	39.6 (42.5, 26–52,10.2)	42.7 (42.0, 28–58,15.0)	51.3 (50.5, 40–62,7.8)	34.0	42.0 (40.0, 36–50, 7.2)	42.8 (44.0, 26–62, 10.5)
Female sex, n (%)	9 (75.0)	3 (100.0)	2 (33.3)	0	1 (33.3)	15 (60.0)
Race, n (%)						
White	10 (83.3)	2 (66.7)	6 (100.0)	1 (100.0)	3 (100.0)	22 (88.0)
Black/African-American	2 (16.7)	1 (33.3)	0	0	0	3 (12.0)
Mean (median, range, SD) BMI, kg/m^2^	30.2 (29.4, 23.4–39.0, 5.4)	40.0 (38.5, 28.7–52.7, 12.1)	31.1 (33.1, 23.7–34.3, 4.1)	29.3	24.7 (25.0, 22.5–26.6, 2.1)	30.9 (29.3, 22.5–52.7, 6.7)
Mean (median, range, SD) pain rating, NRS**	4.6 (5.0, 2–8, 1.7)	4.7 (4.0, 2–8, 3.1)	1.2 (1.0, 0–2, 0.8)	4.0	2.0 (1.0, 0–5, 2.7)	3.4 (3.0, 0–8, 2.3)
Mean (median, range, SD) baseline CRP, mg/dL	4.9 (2.1, 0.8–19.8, 5.8)	0.5 (0.3, 0.1–1.0, 0.4)	0.2 (0.2, 0.1–0.4, 0.1)	1.1	0.1 (0.1, 0.1–0.2, 0.05)	2.5 (0.92, 0.1–19.8, 4.6)
Pericarditis medications, n (%)						
Aspirin	0	0	2 (33.3)	0	0	2 (8.0)
NSAIDs	6 (50.0)	1 (33.3)	4 (66.7)	0	1 (33.3)	12 (48.0)
Colchicine	8 (66.7)	3 (100.0)	6 (100.0)	1 (100.0)	2 (66.7)	20 (80.0)
Corticosteroids	4 (33.3)	2 (66.7)	6 (100.0)	0	3 (100.0)	15 (60.0)
Pericarditis medication categories, n (%)						
0	3 (25.0)	0	0	0	0	3 (12.0)
1	2 (16.7)	0	0	1 (100.0)	0	3 (12.0)
2	5 (41.7)	3 (100.0)	0	0	3 (100.0)	11 (44.0)
≥3	2 (16.7)	0	6 (100.0)	0	0	8 (32.0)
Number of previous pericarditis recurrences						
Mean	1.8	2.0	3.3	8.0	3.3	2.6

*Part 1.

†Part 2.

‡Part 3.

§Part 4.

¶Part 5.

**11-point numeric scale, ranging from zero (0, no pain) to 10 (10, pain as bad as possible).

BMI, body mass index; CRP, C reactive protein; CS-dep, corticosteroid dependent; NRS, numeric rating scale; NSAIDs, nonsteroidal anti-inflammatory drugs; PPS, postpericardiotomy syndrome.;

## Efficacy

Efficacy was assessed using multiple endpoints examining patient-reported pericarditis pain, HRQOL and inflammatory and clinical manifestations of pericarditis.

### Resolution of acute pericarditis episodes

**Figure 2:**
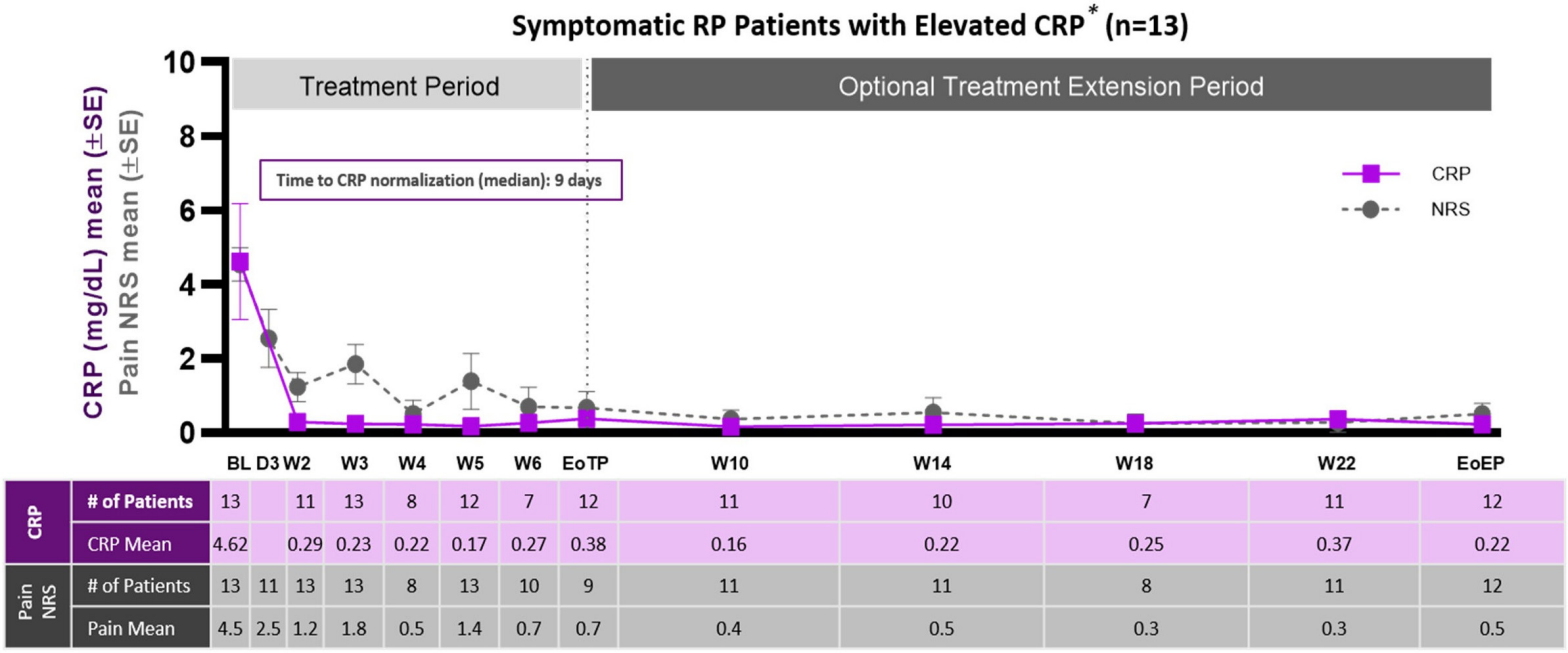
NRS scores (pain) and CRP levels in symptomatic patients with elevated CRP. CRP, C reactive protein; NRS, numeric rating scale; RP, recurrent pericarditis.

In symptomatic patients with elevated CRP >1 mg/dL (n=13) (parts 1 and 4), reductions in average pericarditis pain were observed as soon as after the first (loading) dose of rilonacept, and these decreases were maintained throughout the study (figure 2). Reductions in pain averaged 4 points on an 11-point pain NRS (ranging from 0 to 10). Similarly, decreases in CRP were observed after the first rilonacept dose and were maintained throughout the study (figure 2). The median time to CRP normalisation was 9.0 days. Other pericarditis manifestations resolved in these patients (online supplemental results and online supplemental table 1). For the remaining symptomatic patients (part 2) who had confirmed pericardial inflammation by MRI (n=3), NRS pain reduction was also observed and the low levels of CRP at study entry were maintained.

### Reduced annualised incidence of pericarditis

Across all study parts, there was a reduction in pericarditis episode frequency, as demonstrated by a decrease in mean annualised incidence from 3.9 (SD 3.66) episodes per year for all patients prior to study entry to 0.18 (SD 0.62) in part 1 and 0.0 for the remaining study parts during the study (ie, while on rilonacept treatment) (table 2). Pericarditis recurrence during the study was based on investigator’s judgement. The only on-study pericarditis recurrence occurred in one patient enrolled in part 1 who had a mild episode in the base TP of 5 days’ duration (NRS pain increase from 0 to 2 and CRP of 0.10 mg/dL), which did not require an increase of concomitant therapy nor the addition of a new medication to treat pericarditis; this patient completed the EP without further event.

**Table 2:** Annualised incidence of pericarditis episodes prior to and during the study

Disease status	Idiopathic			PPS	
	Active*	Active†	CS-dep‡	Active§	CS-dep¶
CRP requirement (mg/dL)	>1	≤1	N/A	>1	N/A
N	12	3	6	1	3
Prior to the study**					
Pericarditis episodes per year, mean (median, range, SD)	4.4 (2.4, 0.5–15.0, 4.68)	2.0 (1.0, 1.0–4.0, 1.75)	4.5 (3.4, 1.9–8.6, 2.58)	1.3	3.7 (2.5, 1.5–7.1, 3.02)
**During the study††**					
Patients with pericarditis episodes, n	1‡‡	0	0	0	0
Pericarditis episodes per year, mean (SD)	0.18 (0.62)	0	0	0	0

*Part 1.

†Part 2.

‡Part 3.

§Part 4.

¶Part 5.

**Annualised incidence of pericarditis episodes prior to the study was calculated by dividing the number of episodes (including index, prior recurrences and qualifying episode if applicable) by disease duration in years.

††Annualised incidence of pericarditis episodes during the study was calculated by dividing the number of recurrences during the study by duration of follow-up during the study in years.

‡‡One subject in part 1 presented with increase in pericarditis chest pain from 0 to 2 for 5 days; no medication was added; at visit, CRP was 0.1 mg/dL; it was reported as a pericarditis recurrence by the investigator.

CRP, C reactive protein; CS-dep, corticosteroid dependent; PPS, postpericardiotomy syndrome.;

### Concomitant medication use

Overall, patients were able to stop or reduce the dose of at least one concomitant pericarditis medication without a recurrence (table 3). In particular, of 13 patients who completed the study who were receiving CS at baseline, 11 (84.6%) discontinued CS and the remaining 2 reduced the dose; there were no recurrences in these patients (table 4). Additional information can be found in online supplemental results.

**Table 3:** Summary of pericarditis medications in all patients during base TP and EP

	Medications					
	At least 1	Analgesics	Aspirin	NSAIDs	Colchicine	CS
**Idiopathic, active, CRP >1 mg/dL (part 1), n/N (%)**						
Dose stopped	6/8 (75.0)	0/0	0/0	4/6 (66.7)	2/8 (25.0)	3/3 (100.0)
Dose decreased	1/8 (12.5)	0/0	0/0	1/6 (16.7)	0/8	0/3
Dose increased	1/8 (12.5)	0/0	0/0	0/6	1/8 (12.5)	0/3
Starting new	0/11	0/11	0/11	0/11	0/11	0/11
**Idiopathic, active, CRP ≤1 mg/dL (part 2), n/N (%)**						
Dose stopped	2/3 (66.7)	0/0	0/0	1/1 (100.0)	1/3 (33.3)	1/2 (50.0)
Dose decreased	1/3 (33.3)	0/0	0/0	0/1	0/3	1/2 (50.0)
Dose increased	0/3	0/0	0/0	0/1	0/3	0/2
Starting new	0/3	0/3	0/3	0/3	0/3	0/3
**Idiopathic, CS-dependent (Part 3), n/N (%)**						
Dose stopped	4/5 (80.0)	0/0	0/1	2/4 (50.0)	1/5 (20.0)	4/5 (80.0)
Dose decreased	3/5 (60.0)	0/0	1/1 (100.0)	1/4 (25.0)	1/5 (20.0)	1/5 (20.0)
Dose increased	0/5	0/0	0/1	0/4	0/5	0/5
Starting new	0/5	0/5	0/5	0/5	0/5	0/5
**PPS, active, CRP >1 mg/dL (part 4), n/N (%)**						
Dose stopped	0/1	0/0	0/0	0/0	0/1	0/0
Dose decreased	0/1	0/0	0/0	0/0	0/1	0/0
Dose increased	0/1	0/0	0/0	0/0	0/1	0/0
Starting new	0/1	0/1	0/1	0/1	0/1	0/1
**PPS, CS-dependent (part 5), n/N (%)**						
Dose stopped	3/3 (100.0)	0/0	0/0	0/1	0/2	3/3 (100.0)
Dose decreased	1/3 (33.3)	0/0	0/0	1/1 (100.0)	0/2	0/3
Dose increased	0/3	0/0	0/0	0/1	0/2	0/3
Starting new	0/3	0/3	0/3	0/3	0/3	0/3

CRP, C reactive protein; CS-dependent, corticosteroid dependent; EP, extension period; NSAIDs, non-steroidal anti-inflammatory drugs; PPS, postpericardiotomy syndrome; TP, treatment period.

**Table 4:** Corticosteroid use in all patients

Disease status: CRP requirement (mg/dL): n:	Idiopathic			PPS		Total
	Active* >1 12	Active† ≤1 3	CS-dep‡ N/A 6	Active§ >1 1	CS-dep¶ N/A 3	All*†‡§¶ N/A 25
**Baseline**						
Patients on prednisone, n	4	2	6	0	3	15
Mean dose (mg/day)	8.4	40.0	8.9	0	7.7	12.7
Median dose	10.0	40.0	4.5	0	5.0	8.0
Min	1.0	30.0	2.5	0	3.0	1.0
Max	12.5	50.0	30.0	0	15.0	50.0
SD	5.06	14.14	10.49	NA	6.43	13.72
**Corticosteroid changed during TP and EP combined in patients who completed EP**						
Prednisone dose decreased	0/3	1/2 (50.0)	1/5 (20.0)	0/0	0/3	2/13 (15.4)
Prednisone stopped	3/3 (100.0)	1/2 (50.0)	4/5 (80.0)	0/0	3/3 (100.0)	11/13 (84.6)
Prednisone dose increased	0/3	0/2	0/5	0/0	0/3	0/13
Prednisone initiated	0/11	0/3	0/5	0/1	0/3	0/23

*Part 1.

†Part 2.

‡Part 3.

§Part 4.

¶Part 5.

CRP, C reactive protein; CS-dep, corticosteroid dependent; EP, extension period; PPS, postpericardiotomy syndrome; TP, treatment period.

### Additional endpoints

HR-QOL, as measured by PROMIS questionnaire (V.1.2-Global Health), improved in symptomatic patients with elevated CRP, and findings from the exploratory Cardiac MRI substudy (11 patients) showed improvement of pericardial inflammation (online supplemental results, online supplemental table 2).

## Safety

All patients experienced one or more TEAEs during the study (table 5). The majority of TEAEs (92%) were mild or moderate in severity. The most common AEs were injection site reactions (15/25 patients (60%)), nasopharyngitis, arthralgia and diarrhoea (online supplemental table 3). No systemic hypersensitivity reactions were reported during the study. All injection site reactions were assessed as ‘mild’ by the investigator, and none resulted in treatment discontinuations.

**Table 5:** Overview of TEAEs

Disease status: CRP requirement (mg/dL): n:	Idiopathic			PPS		Total
	Active* >1 12	Active† ≤1 3	CS-dep‡ N/A 6	Active§ >1 1	CS-dep¶ N/A 3	All*†‡§¶ N/A 25
Patients with ≥1 TEAE, n (%)	12 (100.0)	3 (100.0)	6 (100.0)	1 (100.0)	3 (100.0)	25 (100.0)
Patients with ≥1 treatment-related TEAE, n (%)	9 (75.0)	2 (66.7)	3 (50.0)	1 (100.0)	2 (66.7)	17 (68.0)
Patients with ≥1 serious TEAE, n (%)	2 (16.7)	0	0	0	0	2 (8.0)
Patients with ≥1 treatment-related serious TEAE, n (%)	1 (8.3)	0	0	0	0	1 (4.0)
Patients with ≥1 TEAE leading to discontinuation, n (%)	1 (8.3)	0	0	0	0	1 (4.0)
Patients with ≥1 TEAE leading to death, n (%)	0	0	0	0	0	0
Patients with TEAEs by severity, n (%)						
Mild	9 (75.0)	3 (100.0)	4 (66.7)	1 (100.0)	2 (66.7)	19 (76.0)
Moderate	2 (16.7)	0	2 (33.3)	0	0	4 (16.0)
Severe	1 (8.3)	0	0	0	1 (33.3)	2 (8.0)

*Part 1.

†Part 2.

‡Part 3.

§Part 4.

¶Part 5.

CRP, C reactive protein; CS-dep, corticosteroid dependent; PPS, postpericardiotomy syndrome; TEAEs, treatment-emergent adverse events.

Drug-related TEAEs were reported in 17 (68%) patients; 64% were classified as general disorders or administrative site conditions. Two serious TEAEs were reported in patients enrolled in part 1, both of which resolved without sequelae. Rilonacept was discontinued in one patient on concomitant CS with a history of skin infections who developed a serious AE of an SC abscess (cultures positive for *Finegoldia magna*) on the torso that resolved with intravenous antibiotics and surgical incision/drainage; it was reported as a severe AE and was deemed possibly related to study drug by the investigator. The second patient experienced a serious AE of non-cardiac chest pain deemed unrelated to study drug by the investigator; patient continued rilonacept. Increases in total cholesterol, high-density lipoprotein cholesterol, low-density lipoprotein cholesterol and triglycerides levels were observed, as expected, in patients during treatment with rilonacept (online supplemental table 4). None of the patients initiated new lipid-lowering therapy on study. There were no major abnormalities noted in the haematology and chemistry test results during rilonacept treatment.

## Discussion

RP results in significant morbidity, and there are no FDA-approved therapies. This pilot study represents the first use of rilonacept in idiopathic or postpericardiotomy pericarditis. In patients with symptomatic RP with elevated CRP, rilonacept resulted in rapid and sustained reduction in patient-reported pericarditis pain and CRP. Second, annualised recurrences of pericarditis were lower after treatment, and HRQOL improved. Third, prednisone was successfully discontinued in 84.6% of patients receiving prednisone at baseline and was either discontinued or tapered in all CS-dependent patients. Fourth, imaging findings suggestive of pericardial inflammation, as assessed with cardiac MRI or echocardiography, also improved. Therapy was generally well tolerated with only one discontinuation due to a serious AE. Antidrug (antirilonacept) antibodies were detected at at least one timepoint during the study in 14 out of 25 patients; the titres were low in the majority of these patients. Local injection site reactions were reported in 57.1% of ADA-positive patients versus 36.4% in ADA negative patients. Presence of ADAs had minimal impact on rilonacept concentrations.

### Current treatment paradigms

When pericarditis is refractory to NSAIDs and colchicine, current guidelines recommend CS.^2^ Even though CS can provide rapid control of symptoms, notable side effects often occur, which are dose dependent and duration dependent.^2 14 15^ Unfortunately, short CS courses with rapid tapering may exacerbate recurrence,^16–21^ and long durations of CS may be needed to control the disease. In addition, despite protracted courses with gradual tapering, patients often repeatedly recur when the CS dose is decreased below a specific level.^3^ Although alternative immunosuppressive options are sometimes used in patients who require unacceptably high long-term CS doses (azathioprine and intravenous immunoglobulins (IVIG)),^22–24^ data supporting their efficacy are lacking. Therefore, similar to systemic inflammatory diseases, CS-sparing therapies are needed for RP. Our initial results suggest that rilonacept has promise as a CS-sparing therapy.

While previous studies and case reports explored the potential for targeting the IL-1 receptor in RP, these studies with anakinra were limited in the number and characteristics of their patient population, as well as the use of concomitant colchicine.^6 7^ Additionally, there is evidence that targeting of IL-1β alone (eg, with canakinumab) provides insufficient control of the disease and has been associated with relapses.^25 26^ Targeting both IL-1α and IL-1β, as rilonacept does, provides proper control of the disease. Rilonacept offers a rapid treatment response, convenience of weekly dosing and evidence of improved quality of life.

### Study limitations

This study was limited by the single-active-arm, open-label design. Specifically, there was no placebo control group, which may be particularly relevant in the assessment of subjective measures, such as pain scores. However, changes in objective measures such as CRP, pericardial inflammation by cardiac MRI and pericardial effusion by cardiac MRI and echocardiography support efficacy. Nonetheless, comprehensive cardiac imaging with MRI and echocardiography was not mandated in all patients, and there may be a selection bias in patients who underwent these evaluations. Moreover, investigators were encouraged to taper concomitant therapies during the EP, but this approach was not mandated. In particular, the majority of patients were not tapered off colchicine. This practice may reflect data supporting colchicine in RP^27^ and concern for recurrence in this high-risk patient population after conclusion of the study. Finally, the study cohort was heterogeneous and essentially consisted of two groups: patients with RP having an active recurrence and patients with RP who were CS dependent but without active recurrence. This heterogeneity, coupled with the small sample size, limits the strength of our conclusions. However, the rapid resolution of the acute episode, reduction in number of recurrences even while tapering and discontinuing CS and improvement in HRQOL together indicate a true treatment effect of rilonacept rather than spontaneous resolution of the disease. Regardless, the study accomplished our primary objectives, which were to describe preliminary efficacy and safety data in a broader population of RP patients and inform the design of **R**ilonacept in**H**ibition of interleukin-1 **A**lpha and beta for recurrent **P**ericarditis: a pivotal **S**ymptomatology and **O**utcomes stu**DY** (RHAPSODY), the confirmatory double-blind, placebo-controlled randomised withdrawal phase III pivotal study, which evaluated the efficacy and safety of rilonacept treatment in patients with RP.^28 29^

## Conclusion

In summary, this open-label study represents the first use of rilonacept in idiopathic or postpericardiotomy pericarditis. Rilonacept reduced pericardial pain and inflammation, improved objective features of pericarditis and enhanced quality of life. Furthermore, CSs were successfully tapered and discontinued without pericarditis recurrence which, if confirmed in phase III, could indicate the potential for rilonacept to offer a CS-sparing therapeutic option.

**Key messages**

**What is already known on this subject?**

- There are currently no FDA-approved therapies for recurrent pericarditis (RP). While the underlying mechanism driving RP is not completely known, the interleukin (IL) 1 family of cytokines has been implicated in the inappropriate autoinflammatory response and activation of the innate immune system. A recombinant IL-1 receptor antagonist, anakinra, was shown to reduce risk of recurrent events in a small, investigator-initiated, placebo-controlled trial.

**What might this study add?**

- This 6-month study investigated the therapeutic potential of rilonacept, a once-weekly subcutaneously injected IL-1α and IL-1β cytokine trap in patients with both idiopathic and postpericardiotomy syndrome RP and in both acutely symptomatic as well as steroid-dependent clinical presentations.

**How might this impact on clinical practice?**

- This phase II study was an open-label single-active-arm pilot study in a small number of patients to provide scientific evidence supporting the IL-1-mediated pathophysiology of RP and treatment data with rilonacept in different clinical presentations in advance of RHAPSODY, a confirmatory, phase III, double-blind, placebo-controlled study that was recently completed successfully (RHAPSODY; clinicaltrials.gov/NCT03737110). These data support the benefit/risk profile of rilonacept in patients with RP.
