# Supplementary material for: Efficacy and safety of rilonacept for recurrent pericarditis: results from a phase II clinical trial
Source: Heart. 2020 Nov 23;107(6):488–96. doi: 10.1136/heartjnl-2020-317928 (PMC7925818; doi:10.1136/heartjnl-2020-317928)
Supplement: Supplementary data [file heartjnl-2020-317928supp001.pdf]

Supplemental Materials

Supplemental Methods

Exploratory Cardiac Magnetic Resonance Imaging Endpoint

Cardiac magnetic resonance imaging (MRI) at baseline and at 6 months was optional, with the exception of Part 2, in which cardiac MRI was mandatory for eligibility assessment. Cardiac MRI was performed as previously described to assess changes in pericardial delayed hyperenhancement (DHE), as well as effusion size.(1) In brief, DHE images were obtained in long and short-axis orientations ~10 minutes after intravenous injection of Gd-diethylenetriamine penta-acetic acid (0.1-0.2 mmol/kg body weight). Qualitative assessment of pericardial DHE was as follows: none (≤ 50% circumferential DHE at basal, mid and apical ventricular levels), mild (> 50% circumferential DHE at either basal, mid, or apical ventricular levels), moderate (> 50% circumferential DHE at 2 of 3 levels), or severe (> 50% DHE at 3 of 3 levels). Cardiac MRIs were assessed in a core lab blinded to patient data. Both pericardial DHE and pericardial effusion were assessed to provide further objective measures of resolution of pericardial inflammation. For enrollment in part 2, pericardial DHE had to be moderate or severe.

Pharmacokinetic (PK) Assessments

Samples for pharmacokinetic analysis were collected at Screening Visit, day 0, weekly post-treatment for weeks 2 through end of base TP, then monthly during EP. Rilonacept concentrations were quantitated by enzyme-linked immunosorbent assay (ELISA [Regeneron]).

Anti-drug Antibody (ADA) Assessments

Samples for ADA analysis were analyzed at baseline, weeks 2, 3, 4, 6/end of base TP, month 2, and at EP Final Visit. ADAs were detected using a non-quantitative electrochemiluminescent (ECL) bridging immunoassay on the MSD instrument. The bridging assay procedure employs a mouse anti-Rilonacept monoclonal antibody, as positive control, and biotinylated Rilonacept and ruthenium-labeled Rilonacept as bridge components. A tiered risk assessment approach was used to screen, confirm and titer ADAs. Samples were treated with acid to dissociate drug:ADA complexes present in serum samples which allows improved detection of ADA while drug is present in the serum. Neutralizing ani-rilonacept antibodies were not assessed. Positive ADA status was defined as having at least one ADA-positive measurement post-baseline at any time point during the study.

Supplemental Results

Other Pericarditis Manifestations

At the end of the study, other pericarditis manifestations such as pericardial effusion on echocardiogram, ECG changes and pericardial rub also resolved in patients with symptomatic RP of idiopathic or PPS etiology with elevated CRP (Parts 1 and 4): pericardial effusion (6/7 patients), PR depression (2/3 patients), widespread ST elevation (2/2 patients), and pericardial rub (2/2 patients). At the end of the study, two patients were reported to have trivial/physiologic pericardial effusion, and 1 patient had isolated PR depression. (**Supplemental Table 1**).

Changes in Concomitant Medication Use

Overall, out of 20 patients on concomitant pericarditis medications at baseline and who completed the EP, 75% of patients (n=15) successfully stopped, and 30% of patients (n=6) reduced the dose of at least one concomitant pericarditis medication by the end of the study without experiencing a pericarditis recurrence (see **Table 3** in main text).

Tapering and Discontinuation of Corticosteroids and Colchicine

Prednisone (the only CS used in the study for pericarditis) was most frequently discontinued or reduced. A total of 15 patients entered the study with ongoing CS treatment, receiving prednisone for pericarditis at the mean dose 12.7 mg/day (range 1mg-50 mg/day). Of these 15 patients, 13 completed the EP (one patient in Part 1 and one CS-dependent patient in Part 3 did not enter the EP). Of the 13 patients on CS at baseline who completed the study, 11 discontinued prednisone completely (4/5 symptomatic patients, and 7/8 CS-dependent patients) and 2 tapered the dose of CS (1/5 symptomatic patients, and 1/8 CS-dependent patients) without experiencing a recurrence of signs and symptoms of RP while maintaining low average pain and CRP levels (**Supplemental Figure 2).** One of the patients remaining on CS at study end had reduced from 30 to 2.5 mg/day, and the other remained on 30 mg/day (from 50 mg/day), per investigator discretion for disease management during the finite study period. Of the 4 acute symptomatic patients with elevated CRP (Parts 1 and 4) on CS at baseline, 1 patient did not enter the EP; all 3 patients who completed the study stopped their prednisone without disease recurrence while on rilonacept.

In the subset of 9 CS-dependent patients, who were not experiencing acute pericarditis episodes (Parts 3 and 5), 1 patient did not enter EP, but all 8 patients who completed the study either

tapered (1 patient) or stopped (7/8 patients) the CS **(Supplemental Figure 2**) while maintaining low average pain and CRP levels. Of these 9 CS-dependent patients, 2 patients from Part 3 presented with a pericardial effusion on echocardiogram at baseline. Pericardial effusion resolved in one patient and was assessed as trivial/physiologic in another patient at the end of the study.

In the subset of 6 CS-failure patients (patients experiencing an acute pericarditis episode at baseline while receiving CS and colchicine), 5 patients entered the EP; 4 of these patients discontinued CS during the EP.

Overall, the CS-sparing effect of rilonacept was consistent among patients with and without active recurrence at the time of enrollment, and no recurrences were observed (see **Table 4** in main text).

Of 23 patients who completed the EP, 7 (30%) were experiencing an active episode while being treated with colchicine and not corticosteroids at baseline (colchicine-failure patients). One of these seven colchicine-failure patients discontinued colchicine use during the study.

Efficacy in Corticosteroid-Failure and Colchicine-Failure Patients

Of 15 patients who entered the study with ongoing CS treatment, 9 were CS-dependent, and 6 were experiencing an active episode at baseline while also receiving colchicine (CS-failure patients). CS-failure patients experienced rapid and sustained reductions in pericarditis pain and CRP with rilonacept treatment (**Supplemental Figure 3**).

The 7 patients who were experiencing an active episode at baseline while being treated with colchicine and not corticosteroids (colchicine-failure patients) experienced rapid and sustained reductions in pericarditis pain and CRP with initiation of rilonacept (**Supplemental Figure 4**).

Health-Related Quality of Life

A consistent pattern of increased PROMIS scores reflected improvement in HRQOL with rilonacept treatment (**Supplemental Table 2**). At baseline, mean Physical and Mental Global Health scores across all patients were below 50, which is the mean score for the general US population, indicating impaired QOL in symptomatic RP patients as well as CS-dependent patients without active pericarditis. In symptomatic patients of idiopathic (Parts 1 and 2) or PPS etiology (Part 4), the mean baseline Physical and Mental Global Health scores were 39.9 and 44.5, respectively, and improved to 51.3 and 50.5 at the Final Visit. In CS-dependent patients with RP (Parts 3 and 5), the mean baseline Physical and Mental Global Health scores were 43.3 and 46.5, respectively, and improved to 46.8 and 50.7, respectively, at the Final Visit. In addition, improvements in HRQOL were observed after the first 6 weeks of rilonacept treatment (**Supplemental Table 2**).

Exploratory Cardiac Magnetic Resonance Imaging Outcomes

Of 25 study patients, 11 had cardiac MRI at baseline and the Final Visit, including 6 patients with active idiopathic RP and 5 CS-dependent non-active RP patients (4 idiopathic, 1 PPS). Among 8 patients with baseline pericardial DHE (mild, moderate, or severe) and follow-up MRI, DHE improved or resolved in 6 patients. For the 2 patients in which pericardial DHE was not changed, 1 patient (Part 2) had moderate pericardial DHE at baseline and the Final Visit, and the

second patient (Part 5) had mild pericardial DHE at baseline and the Final Visit. One patient (Part 3) had no pericardial DHE at baseline and had mild DHE at the Final Visit. Improvements in DHE were associated with decreases or maintenance of low pain and CRP levels despite discontinuation of CS or reduction in dose.

Patient Enrolled Twice

The case study of the patient who was re-treated with rilonacept for RP provides an example of the persistence and severity of RP as well as the efficacy and tolerability of rilonacept upon re-treatment. This patient with severe RP participated in the study twice, having been enrolled in the study a second time with a recurrence of pericarditis approximately 4.5 months after successfully completing 6 months of rilonacept in her first participation in the trial. Retreatment with rilonacept resulted in a similar clinically meaningful response with similar tolerability, suggesting that the disease improvements represent a true response to treatment rather than a spontaneous improvement due to natural history of the disease. In addition, although limited to one patient, this example provides a framework for evaluating the efficacy and safety of repetitive use of rilonacept in RP.

PK Assessments

A total of 25 unique patients provided 211 samples for pharmacokinetic analysis. The mean rilonacept concentration time profile demonstrated moderate to high variability (30.1-69.2%CV) between patients across the duration of the study. Following the loading dose (320 mg), no

additional accumulation of rilonacept was observed, and the median trough concentration over the base TP and EP was approximately 30,000 ng/mL (**Supplemental Figure 5**).

Immunogenicity

In total, among 25 unique patients, 14 (56%) were classified as positive for ADA (anti-drug [rilonacept] antibodies), i.e., ADA detected in at least one post-baseline serum sample during the study. Two of these 14 patients had low titers (1:50) of pre-existing ADAs detected before administration of rilonacept and continued to have detectable low titers of ADAs (up to 1:150) during the remainder of the study. At Final Visit, 4 patients (17.4%) had detectable ADAs. The majority of patients (12 out of 14) with ADA positivity during the study had low titers (up to 1:150). Among the 14 patients who had ADAs detected at at least 1 post-baseline timepoint, 8 (57.1%) subjects had a TEAE of local injection site reaction. Among the 11 subjects negative for ADAs throughout the study, 4 (36.4%) subjects experienced injection site reactions. Presence of ADAs had a minimal impact on rilonacept trough concentrations (**Supplemental Figure 6**).

REFERENCES

1. Cremer PC, Tariq MU, Karwa A, et al. Quantitative assessment of pericardial delayed hyperenhancement predicts clinical improvement in patients with constrictive pericarditis treated with anti-inflammatory therapy. Circ Cardiovasc Imaging 2015;8(5) doi: 10.1161/CIRCIMAGING.114.003125 [published Online First: 2015/04/24]

Supplemental Figures

Supplemental Figure 1. Patient Disposition

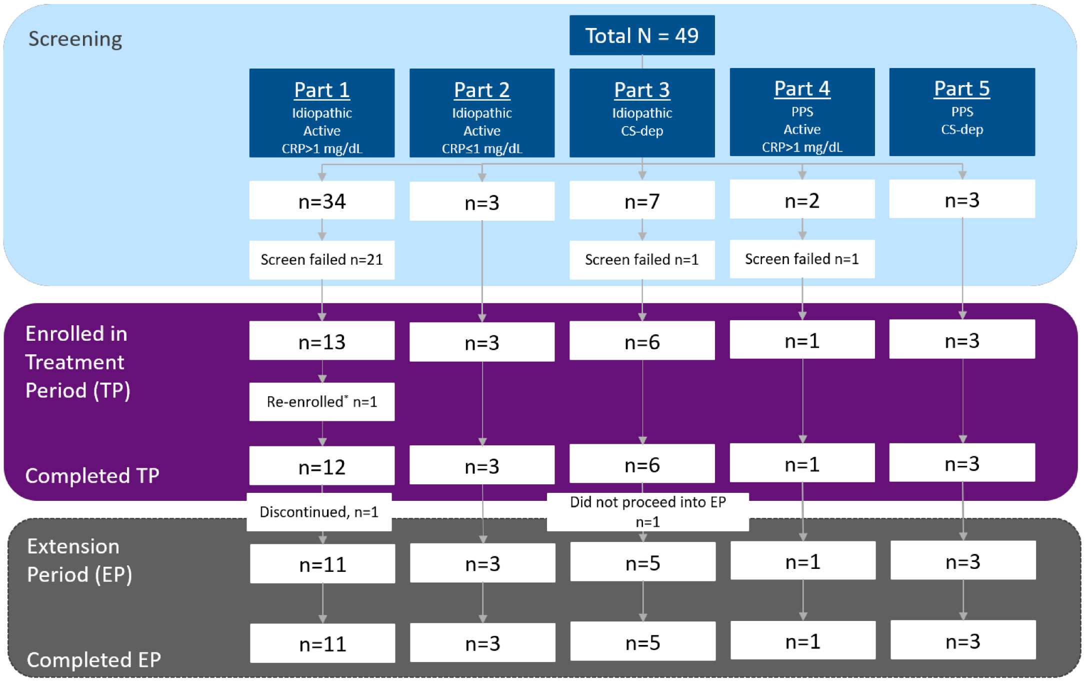

Supplemental Figure 1. Patient disposition.

Among 49 patients screened, 25 unique adult patients were enrolled and received rilonacept. One patient participated in the study twice, but only assessments from this patient's first participation are included in the analysis. Of 23 patients who entered the EP, 23 (100%) completed it; one CS-dependent PPS patient (Part 3) completed the base TP but declined to continue into the EP, and one symptomatic idiopathic RP patient (Part 1) experienced a serious AE and discontinued the study drug after Visit 4 in the base TP.

*One patient participated in the study twice (N=26); however, data are reported for 25 unique patients.

CRP, C-reactive protein; CS-dep, corticosteroid dependent; EP, extension period; PPS, post-pericardiotomy syndrome; TP, treatment period.

Supplemental Figure 2. NRS Scores (Pain) and CRP Levels in Non-active CS-dependent Patients[a]

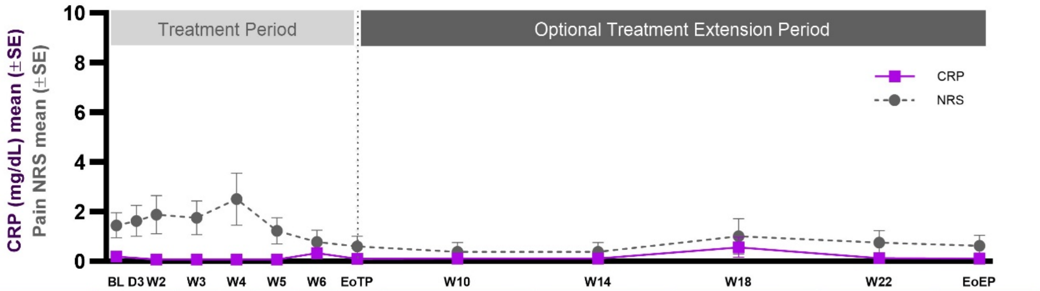

		BL	D3	W2	W3	W4	W5	W6	EoTP	W10	W14	W18	W22	EoEP
# Patients on CS		9		9	9	9	9	9	9	8	8	2	1	1
CRP	# of Patients	9		8	9	5	9	5	9	7	8	4	8	8
	CRP Mean	0.19		0.07	0.07	0.07	0.07	0.33	0.09	0.10	0.11	0.56	0.12	0.12
Pain NRS	# of Patients	9	8	8	8	4	9	9	5	8	8	4	8	8
	Pain Mean	1.4	1.6	1.9	1.8	2.5	1.2	0.8	0.6	0.4	0.4	1.0	0.8	0.6

Supplemental Figure 2. NRS scores (pain) and CRP levels. Parts 3 and 5: Corticosteroid-dependent patients

The subset of 9 CS-dependent patients who were not experiencing acute pericarditis episodes (Parts 3 and 5) maintained low average pain and CRP levels.

[a]Part 3 and Part 5 combined

BL, baseline; CRP, C-reactive protein; CS-dep, corticosteroid dependent; D, day; EP, extension period; NRS, Numeric Rating Scale; RP, recurrent pericarditis; SE, standard error; TP, treatment period; W, week. The number of patients with CRP and pain data changes over time due to combining the visits for some patients and not all patients having assessments at all visits.

Supplemental Figure 3. NRS Scores (Pain) and CRP Levels in CS-failure Patients (n=6)[a]

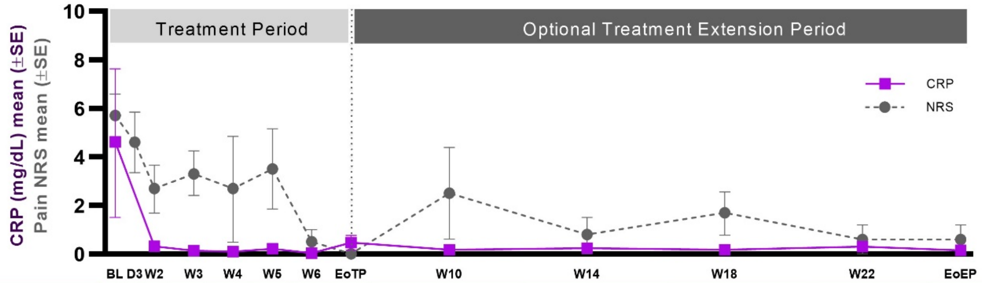

		BL	D3	W2	W3	W4	W5	W6	EoTP	W10	W14	W18	W22	EoEP
# Patients on CS		**6**	**6**	**6**	**6**	**5**	**5**	**5**	**5**	**3**	**2**	**1**	**1**	**1**
CRP	# of Patients	6		6	6	3	5	1	5	4	3	3	5	5
	CRP Mean	4.6		0.3	0.1	0.1	0.2	0.03	0.5	0.2	0.2	0.2	0.3	0.1
Pain NRS	# of Patients	6	5	6	6	3	6	4	3	4	4	3	5	5
	Pain Mean	5.7	4.6	2.7	3.3	2.7	3.5	0.5	0.0	2.5	0.8	1.7	0.6	0.6

Supplemental Figure 3. NRS scores (pain) and CRP levels in Corticosteroid-failure Patients

The subset of 6 CS-failure patients who were experiencing an acute pericarditis episode despite treatment with corticosteroids at baseline experienced rapid and sustained reductions in pericarditis pain and CRP with initiation of rilonacept.

[a]Patients with an active pericarditis episode on CS at enrollment (safety population); one CS failure patient discontinued during the TP due to an SAE, and the resulting subgroup that completed the EP (EP population; n=5) had similar results

BL, baseline; CRP, C-reactive protein; D, day; EoTP, end of treatment period; EoEP, end of extension period; EP, extension period; NRS, Numeric Rating Scale; RP, recurrent pericarditis; SE, standard error; TP, treatment period; W, week. The number of patients with CRP and pain data changes over time due to combining the visits for some patients and not all patients having assessments at all visits.

Supplemental Figure 4. NRS Scores (Pain) and CRP Levels in Colchicine-failure Patients

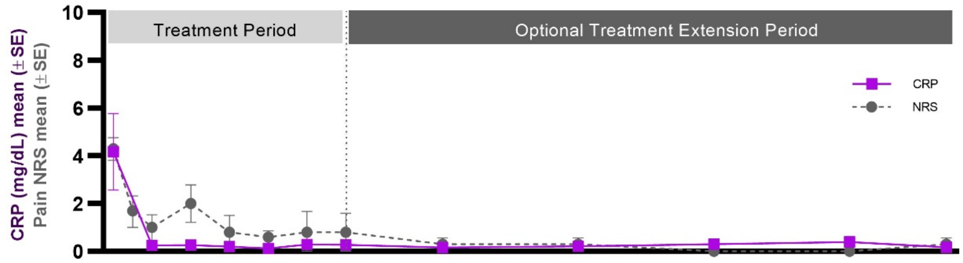

		BL	D3	W2	W3	W4	W5	W6	EoTP	W10	W14	W18	W22	EoEP
**# Patients on Colchicine**		**7**	**6**	**6**	**6**	**6**	**6**	**6**	**6**	**6**	**6**	**6**	**6**	**6**
CRP	# of Patients	7		7	7	4	7	6	5	7	7	4	6	7
	CRP Mean	4.2		0.3	0.3	0.2	0.1	0.3	0.3	0.2	0.2	0.3	0.4	0.2
Pain NRS	# of Patients	7	6	7	7	4	7	6	5	7	7	5	6	7
	Pain Mean	4.3	1.7	1.0	2.0	0.8	0.6	0.8	0.8	0.3	0.3	0.0	0.0	0.3

Supplemental Figure 4. NRS scores (pain) and CRP levels in Colchicine-failure Patients

The subset of 7 colchicine-failure patients who were experiencing an acute pericarditis episode despite treatment at baseline with colchicine but not corticosteroids experienced rapid and sustained reductions in pericarditis pain and CRP with initiation of rilonacept.

[a]Patients with an active pericarditis episode on colchicine and not CS at enrollment (safety population)

BL, baseline; CRP, C-reactive protein; D, day; EoTP, end of treatment period; EoEP, end of extension period; EP, extension period; NRS, Numeric Rating Scale; RP, recurrent pericarditis; SE, standard error; TP, treatment period; W, week. The number of patients with CRP and pain data changes over time due to combining the visits for some patients and not all patients having assessments at all visits.

Supplemental Figure 5 Mean Rilonacept Concentration Time Profile

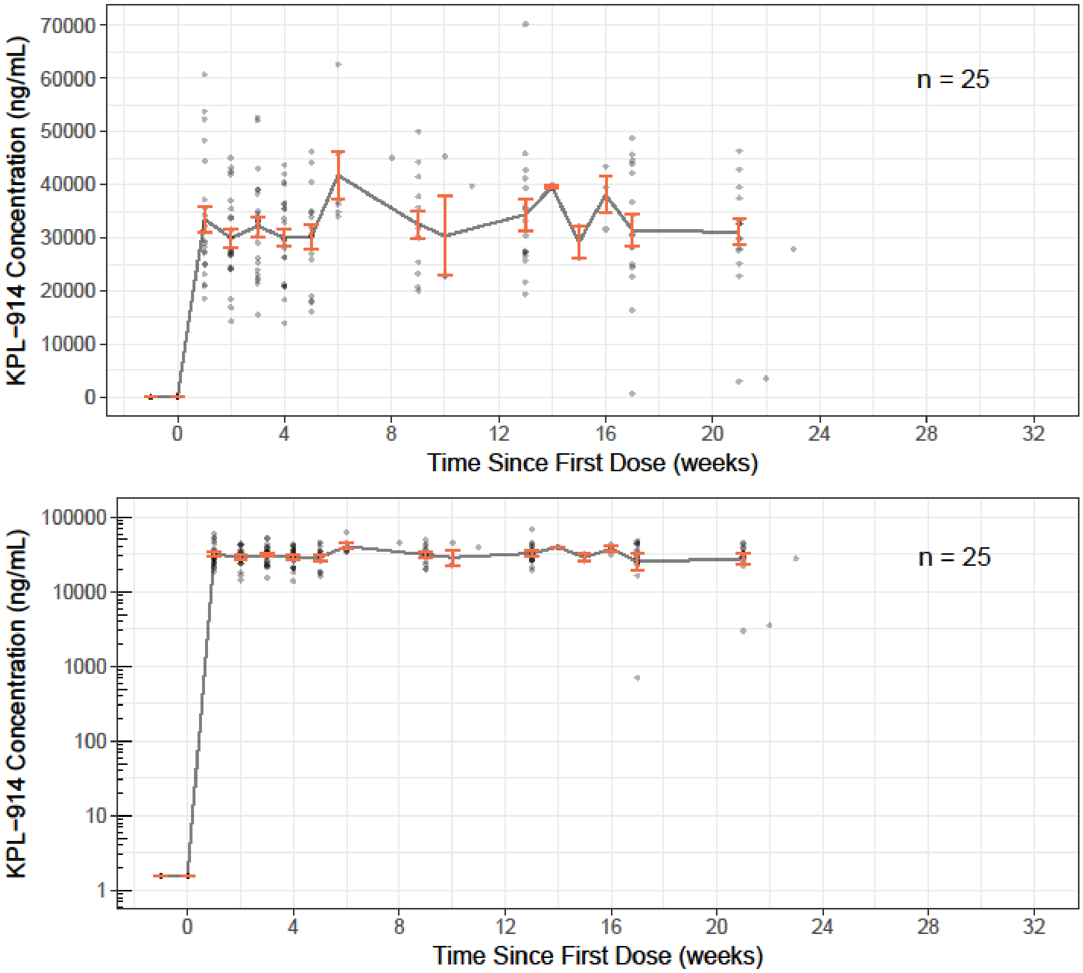

Supplemental Figure 5. Mean rilonacept concentration time profile**.**

Mean rilonacept concentration time profile demonstrated moderate to high variability (30.1-69.2%CV) between patients across the duration of the study. Following the loading dose (320 mg), no additional accumulation of rilonacept was observed, and the median trough concentration over the base TP and EP was approximately 30,000 ng/mL

Supplemental Figure 6 Mean Rilonacept Concentration Time Profile by ADA Status

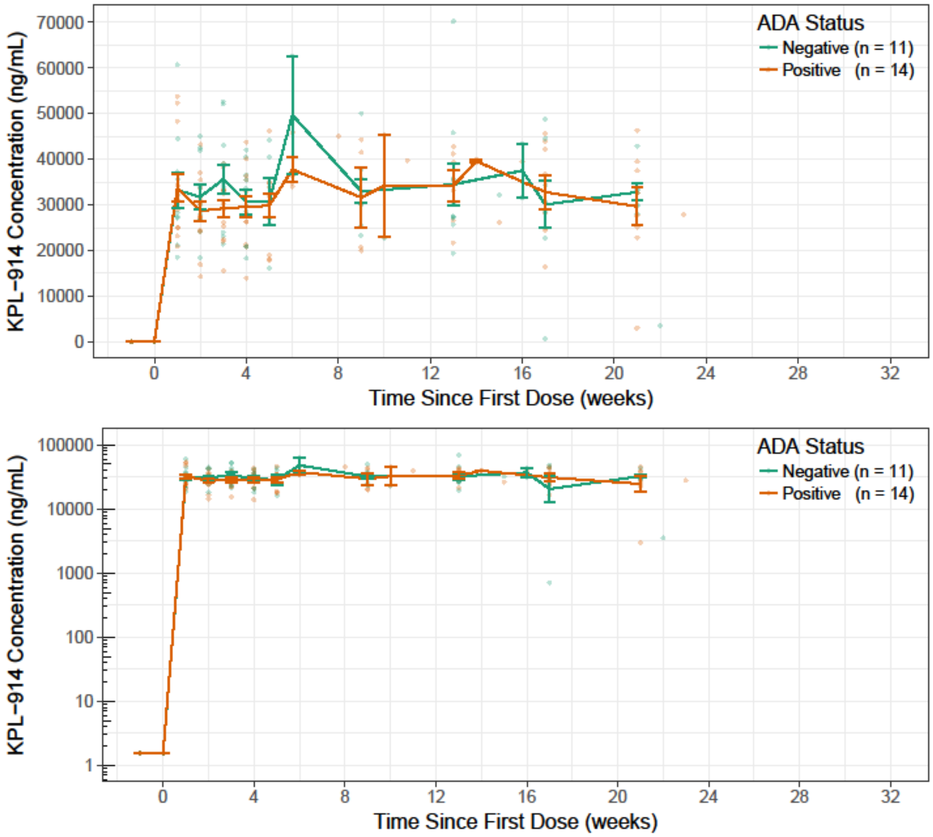

Supplemental Figure 6. Mean rilonacept concentration time profile by ADA status. ADA, anti-drug antibody

Presence of ADAs had a minimal impact on rilonacept trough concentrations.

Supplemental Tables

Supplemental Table 1. Improvement in Pericarditis Symptomatology with Rilonacept

	Idiopathic			PPS		Idiopathic and PPS
**Disease status: CRP requirement (mg/dL): n:**	**Active[*] >1 12**	**Active[†] ≤1 3**	**CS-dep[‡] N/A 6**	**Active[§] >1 1**	**CS-dep[ǁ] N/A 3**	**Active[*,§] >1 13**
Baseline, n (%)						
Widespread ST-segment elevation	2/12 (16.7)	0/3	0/6	0/1	0/3	2/13 (15.4)
PR-segment depression	3/12 (25.0)	0/3	0/6	0/1	0/3	3/13 (23.1)
Pericardial rub	2/12 (16.7)	0/3	0/6	0/1	0/3	2/13 (15.4)
Fever	0/12	0/3	0/6	0/1	0/3	0/13
Pericardial effusion on ECHO	7/12 (58.3)	0/3	2/6 (33.3)	0/1	0/3	7/13 (53.8)
End of TP (Visit 7), n (%)						
Widespread ST-segment elevation	0/12	0/2	0/6	0/1	0/3	0/13
PR-segment depression	1/12 (8.3)	0/2	0/6	0/1	0/3	1/13 (7.7)
Pericardial rub	0/11	0/3	0/6	0/1	0/3	0/12
Fever	0/12	0/3	0/6	0/1	0/3	0/13
Pericardial effusion on ECHO	1/12 (8.3)	0/2	1/6 (16.7)	0/1	0/3	1/13 (7.7)
Final Visit, n (%)						
Widespread ST-segment elevation	0/11	0/3	0/5	0/1	0/3	0/12

PR-segment depression	1/11 (9.1)	0/3	0/5	0/1	0/3	1/12 (8.3)
Pericardial rub	0/11	0/3	0/5	0/1	0/3	0/12
Fever	0/11	0/3	0/5	0/1	0/3	0/12
Pericardial effusion on ECHO	1[#]/11 (9.1)	0/3	1/5 (20.0)	0/1	0/3	1/12 (8.3)

[*]Part 1; [†]Part 2; [‡]Part 3; [§]Part 4; [‖]Part 5; [#]Patient with effusion at baseline, no effusion at EoT Visit and trivial effusion (not pathological) at Final Visit
CRP, C-reactive protein; CS-dep, corticosteroid-dependent; ECHO, echocardiography; PPS, post-pericardiotomy syndrome; TP, treatment period.

Supplemental Table 2: Improvements in PROMIS[*] Global Health, v1.2 questionnaire scores

	Idiopathic or PPS	
	**Active[†] (n=16)**	**CS-dependent[‡] (n=9)**
**Global Physical Health, n, mean (Median, Range, SD)**		
Baseline	16, 39.94 (41.05, 23.50-54.10, 8.941)	7, 43.3 (42.30, 37.40-54.10, 5.311)
End of TP	15, 51.35 (50.80. 34.90-61.90,7.962)	9, 45.09 (44.90, 39.80-54.10, 4.057)
Final Visit	15, 51.32 (54.10, 39.80-61.90, 6.564)	8, 46.81 (47.70, 26.70-57.70, 9.266)
**Global Mental Health, n, mean (Median, Range, SD)**		
Baseline	16, 44.5 (48.30, 25.10-62.50, 10.484)	7, 46.49 (43.50, 38.80-59.00, 7.767)
End of TP	15, 50.13 (50.80, 28.40-67.60, 11.325)	9, 47.91 (45.80, 43.50-59.00, 5.509)
Final Visit	15, 50.54 (53.30, 28.40-67.60, 10.995)	8, 50.66 (50.80, 41.10, 59.00, 6.299)

[*]PROMIS® - Patient Reported Outcomes Measurement Information System. The higher the score, the better global health is. US national average score for Global Physical and Mental Health is 50 (SD 10); [†]Part 1, 2, and 4; [‡]Part 3 and 5
CS-dependent, corticosteroid dependent; PPS, post-pericardiotomy syndrome; SD, standard deviation; TP, treatment period.

Supplemental Table 3. TEAEs by System Organ Class[*]

Disease status:	Idiopathic			PPS		Total
CRP requirement (mg/dL):<br>n:	Active[†]<br>>1<br>12	Active[‡]<br>≤1<br>3	CS-dep[§]<br>N/A<br>6	Active[‖]<br>>1<br>1	CS-dep[#]<br>N/A<br>3	All[†,‡,§,‖,#]<br>N/A<br>25
Number of patients with ≥1 TEAE	12 (100.0)	3 (100.0)	6 (100.0)	1 (100.0)	3 (100.0)	25 (100.0)
General disorders and administration site conditions	6 (50.0)	2 (66.7)	4 (66.7)	1 (100.0)	3 (100.0)	16 (64.0)
Injection site reaction	1 (8.3)	0	2 (33.3)	1 (100.0)	2 (66.7)	6 (24.0)
Fatigue	0	0	1 (16.7)	0	1 (33.3)	2 (8.0)
Injection site bruising	1 (8.3)	0	0	1 (100.0)	0	2 (8.0)
Injection site erythema	1 (8.3)	1 (33.3)	0	0	0	2 (8.0)
Injection site pain	1 (8.3)	0	1 (16.7)	0	0	2 (8.0)
Non-cardiac chest pain	1 (8.3)	0	1 (16.7)	0	0	2 (8.0)
Peripheral swelling	0	1 (33.3)	0	0	1 (33.3)	2 (8.0)
Application site bruise	1 (8.3)	0	0	0	0	1 (4.0)
Application site erythema	1 (8.3)	0	0	0	0	1 (4.0)
Chest discomfort	1 (8.3)	0	0	0	0	1 (4.0)
Injection site joint warmth	1 (8.3)	0	0	0	0	1 (4.0)
Pyrexia	1 (8.3)	0	0	0	0	1 (4.0)
Ulcer hemorrhage	0	0	0	1 (100.0)	0	1 (4.0)
Musculoskeletal and connective tissue disorders	3 (25.0)	0	4 (66.7)	1 (100.0)	2 (66.7)	10 (40.0)
Arthralgia	0	0	2 (33.3)	0	1 (33.3)	3 (12.0)
Musculoskeletal pain	1 (8.3)	0	1 (16.7)	0	0	2 (8.0)
Limb discomfort	1 (8.3)	0	0	0	0	1 (4.0)
Muscle twitching	1 (8.3)	0	0	0	0	1 (4.0)
Musculoskeletal chest pain	0	0	0	0	1 (33.3)	1 (4.0)
Neck pain	0	0	0	1 (100.0)	0	1 (4.0)
Pain in extremity	0	0	1 (16.7)	0	0	1 (4.0)
Infections and infestations	5 (41.7)	1 (33.3)	1 (16.7)	0	1 (33.3)	8 (32.0)
Nasopharyngitis	3 (25.0)	0	1 (16.7)	0	0	4 (16.0)
Cellulitis	2 (16.7)	0	0	0	0	2 (8.0)
Sinusitis	0	1 (33.3)	0	0	0	1 (4.0)
Subcutaneous abscess	1 (8.3)	0	0	0	0	1 (4.0)

Upper respiratory tract infection	0	0	0	0	1 (33.3)	1 (4.0)
Urinary tract infection	1 (8.3)	0	0	0	0	1 (4.0)
Gastrointestinal disorders	6 (50.0)	0	0	0	0	6 (24.0)
Diarrhoea	3 (25.0)	0	0	0	0	3 (12.0)
Dyspepsia	1 (8.3)	0	0	0	0	1 (4.0)
Haemorrhoids	1 (8.3)	0	0	0	0	1 (4.0)
Nausea	1 (8.3)	0	0	0	0	1 (4.0)
Toothache	1 (8.3)	0	0	0	0	1 (4.0)
Investigations	2 (16.7)	0	3 (50.0)	1 (100.0)	0	6 (24.0)
Blood cholesterol increased	1 (8.3)	0	1 (16.7)	0	0	2 (8.0)
Blood creatine phosphokinase increased	1 (8.3)	0	0	1 (100.0)	0	2 (8.0)
Liver function test increased	2 (16.7)	0	0	0	0	2 (8.0)
Alanine aminotransferase increased	0	0	1 (16.7)	0	0	1 (4.0)
Aspartate aminotransferase increased	0	0	1 (16.7)	0	0	1 (4.0)
C-reactive protein increased	0	0	0	1 (100.0)	0	1 (4.0)
Hepatic enzyme increased	0	0	1 (16.7)	0	0	1 (4.0)
High density lipoprotein increased	1 (8.3)	0	0	0	0	1 (4.0)
Lipids increased	0	0	0	1 (100.0)	0	1 (4.0)
Weight increased	0	0	0	1 (100.0)	0	1 (4.0)
Respiratory, thoracic and mediastinal disorders	0	1 (33.3)	2 (33.3)	0	0	3 (12.0)
Cough	0	0	1 (16.7)	0	0	1 (4.0)
Dyspnoea	0	0	1 (16.7)	0	0	1 (4.0)
Dyspnoea at rest	0	0	1 (16.7)	0	0	1 (4.0)
Painful respiration	0	0	1 (16.7)	0	0	1 (4.0)
Productive cough	0	1 (33.3)	0	0	0	1 (4.0)
Skin and subcutaneous tissue disorders	0	0	1 (16.7)	1 (100.0)	1 (33.3)	3 (12.0)
Erythema	0	0	0	0	1 (33.3)	1 (4.0)
Rash	0	0	1 (16.7)	0	0	1 (4.0)

Skin ulcer	0	0	0	1 (100.0)	0	1 (4.0)
Cardiac disorders	0	1 (33.3)	1 (16.7)	0	0	2 (8.0)
Angina pectoris	0	1 (33.3)	0	0	0	1 (4.0)
Cardiac discomfort	0	0	1 (16.7)	0	0	1 (4.0)
Pericarditis	0	0	1 (16.7)	0	0	1 (4.0)
Ear and labyrinth disorders	2 (16.7)	0	0	0	0	2 (8.0)
Vertigo	1 (8.3)	0	0	0	0	1 (4.0)
Vertigo positional	1 (8.3)	0	0	0	0	1 (4.0)
Nervous system disorders	1 (8.3)	0	1 (16.7)	0	0	2 (8.0)
Headache	1 (8.3)	0	1 (16.7)	0	0	2 (8.0)
Eye disorders	1 (8.3)	0	0	0	0	1 (4.0)
Dry eye	1 (8.3)	0	0	0	0	1 (4.0)
Injury, poisoning and procedural complications	1 (8.3)	0	0	0	0	1 (4.0)
Post procedural discharge	1 (8.3)	0	0	0	0	1 (4.0)
Metabolism and nutrition disorders	0	0	0	1 (100.0)	0	1 (4.0)
Increased appetite	0	0	0	1 (100.0)	0	1 (4.0)

[*]All investigator adverse event terms are coded using MedDRA dictionary version 20.1; [†]Part 1; [‡]Part 2; [§]Part 3; [‖]Part 4; [#]Part 5;
Note: Patients are counted only once within each system organ class and preferred term.
MedDRA, Medical Dictionary for Regulatory Activities; PPS, post-pericardiotomy syndrome; TEAEs, treatment-emergent adverse events; CS-dep, corticosteroid-dependent; CRP, C-reactive protein.

Supplemental Table 4. Summary of Lipid Changes*

	Idiopathic			PPS		Total
**Disease status: CRP requirement (mg/dL): n:**	**Active[*] >1 12**	**Active[†] ≤1 3**	**CS-dep[‡] N/A 6**	**Active[§] >1 1**	**CS-dep[‖] N/A 3**	**All[*,†,‡,§,‖] N/A 25**
Cholesterol (mg/dL)						
Mean at baseline [n]	172.4 [11]	256.0 [1]	203.8 [5]	[0]	174.5 [2]	185.3 [19]
Mean at Final Visit [n]	206.1 [11]	231.0 [3]	213.0 [5]	195.0 [1]	175.0 [3]	206.3 [23]
HDL cholesterol (mg/dL)						
Mean at baseline [n]	45.9 [11]	64.0 [1]	55.6 [5]	[0]	50.0 [2]	49.8 [19]
Mean at Final Visit [n]	56.6 [11]	70.0 [3]	50.4 [5]	43.0 [1]	43.7 [3]	54.7 [23]
LDL cholesterol (mg/dL)						
Mean at baseline [n]	107.6 [11]	147.0 [1]	127.0 [5]	[0]	102.0 [2]	114.2 [19]
Mean at Final Visit [n]	130.5 [11]	138.7 [3]	138.2 [5]	124.0 [1]	98.3 [3]	128.7 [23]
Triglycerides (mg/dL)						
Mean at baseline [n]	116.8 [11]	130.0 [1]	156.4 [5]	[0]	172.0 [2]	133.7 [19]
Mean at Final Visit [n]	133.8 [11]	157.0 [3]	199.8 [5]	229.0 [1]	165.0 [3]	159.4 [23]

*Lipids were measured under fasting and non-fasting conditions.
[*]Part 1; [†]Part 2; [‡]Part 3; [§]Part 4; [‖]Part 5CRP, C-reactive protein; CS-dep, corticosteroid-dependent; HDL, high-density lipoprotein; LDL, low-density lipoprotein; TP, treatment period; PPS, post-pericardiotomy syndrome
